# Influence of empathy on work alienation among Chinese nurses during the COVID-19 pandemic: The mediating effect of ego depletion

**DOI:** 10.3389/fpsyg.2023.1057460

**Published:** 2023-02-02

**Authors:** Yi Cui, Tianqi Yang, Man Zhang, Na Liu, Qin Liu, Lanfang Zhang, Lihua Zhang, Haoshuang Yang, Yinling Zhang

**Affiliations:** ^1^Department of Nursing, Air Force Medical University, Xi’an, China; ^2^Department of Military Medical Psychology, Air Force Medical University, Xi’an, China

**Keywords:** empathy, ego depletion, work alienation, mediating effect, Chinese nurses, COVID-19

## Abstract

**Background:**

Nurses’ work alienation has become increasingly serious due to the increase in workload and risk during the coronavirus disease 2019 (COVID-19). However, no studies have investigated the link between empathy, ego depletion, and work alienation among Chinese nurses. The present study aimed to evaluate Chinese nurses’ empathy, ego depletion, and work alienation and to examine whether nurses’ ego depletion mediates the relationship between empathy and work alienation.

**Methods:**

This was a descriptive, cross-sectional study involving 353 nurses from Shaanxi. The Jefferson Scale of Empathy-Health Professionals, Self-Regulating Fatigue Scale and Work Alienation Questionnaire were used to collect data through an online survey. Structural equation modeling was conducted to analyze the mediating model.

**Results:**

Work alienation was negatively correlated with empathy (*r* = −0.305, *p* < 0.01) and positively correlated with ego depletion (*r* = 0.652, *p* < 0.01). Empathy was negatively correlated with ego depletion (*r* = −0.325, *p* < 0.01). Empathy can directly predict work alienation (*β* = −0.263, *p* < 0.01), while ego depletion has a mediating effect between empathy and work alienation (*β* = −0.309, *p* < 0.01), and the mediating effect accounts for 54.02% of the total effect.

**Conclusion:**

Nurses’ work alienation was at a moderate-to-high level. Improving empathy can reduce work alienation through less ego depletion. Nursing managers should discover nurses’ work alienation as soon as possible. Interventions to improve empathy can help replenish nurses’ psychological resources, thereby reducing ego depletion and work alienation.

## 1. Introduction

The outbreak of COVID-19 exposed the global shortage of human resources for health, in which the demand for nursing staff is unprecedented. According to the World Nursing Status Report released by the World Health Organization in 2020, there is a serious shortage of nursing staff around the world, and there will be a shortage of 4.6 million nurses worldwide by 2030 if no action is taken (World Health Organization, 2020). This reminds us of the importance of expanding the team of nurses, speeding up the development of nursing and improving the nursing system. However, during the COVID-19 pandemic, it is easy for nurses to experience work alienation because they are at increased risk of infection and bear a heavy workload, which is not always understood by the public; furthermore, salaries and respect do not always reflect the proper status of nurses among other professionals, which leads to the loss of nursing staff and the instability of the nursing team (Çınar et al., 2021). Therefore, it is of great significance to explore the mechanism and associated influencing factors of nurses’ work alienation to prevent them from leaving the profession and to continue to meet the growing demand for health services.

### 1.1. Nurses’ work alienation and empathy

Work alienation refers to the subjective psychological experience in which the material, spiritual and other important needs of individuals cannot be met at work, which leads to the separation of employees from work and negative coping at work (Amarat et al., 2019). The sense of work alienation can easily lead to a decline in nurses’ work autonomy (Tummers and Den Dulk, 2013), participation in decision-making (Mudallal et al., 2017), and nursing quality (Jun et al., 2021) and an increase in the turnover rate and nursing safety (Bateman et al., 2020). Especially during the COVID-19 pandemic, the number of nurses had difficulty coping with large-scale infections, and the work pressure increased (Denning et al., 2021). The overloaded workload and serious pandemic also caused a huge psychological impact on nurses, who were prone to different degrees of psychological problems, resulting in work alienation. Although few studies have directly proven that work alienation is related to empathy, previous studies have shown that empathy can lower professional burnout and turnover intention, both of which are related to work alienation (Cheng et al., 2020). Therefore, we speculate that empathy is a crucial factor affecting work alienation.

Empathy in nurses refers to their ability to fully consider the emotional, psychological, and behavioral needs of patients and to meet those needs with respect, care, and warmth that not only addresses their physical needs but also alleviates psychological pain (Wang et al., 2020). A high level of empathy can adjust patients’ mood states and improve nursing quality and clinical nursing satisfaction (Marilaf Caro et al., 2017). At the same time, it can also enhance nurses’ sense of professional benefit, replenish psychological energy, and reduce work burnout. In current clinical nursing, nurses are also infected with COVID-19 like patients and empathize with patients. Nurses can identify and meet the needs of patients more accurately, give more effective care to patients, help them recover health, and experience the value of nursing work better (Hofmeyer and Taylor, 2021). Empathy with patients provides nurses with an intrinsic reward of happiness, which reduces dissatisfaction with nursing work and work alienation (Dor et al., 2019). Hence, we proposed Hypothesis 1: Empathy has a significant association with the work alienation of nurses.

### 1.2. The mediating effect of ego depletion

Based on the theory of limited self-control resources, ego depletion, also known as self-regulating fatigue, refers to the limited consumption of psychological resources caused by an individual performing self-regulating behavior, which may lead to a series of cognitive, emotional, and behavioral problems (Baumeister et al., 1998). The theory of limited self-control resources starts from the perspective of energy, focusing on the impact of the occurrence of the control process on other behaviors, and believes that individual self-control is a limited resource. When this limited resource is used to change previous habits, the individual will consume self-control energy, resulting ego depletion, and ego depletion will lead to subsequent behavior failure or reduced efficiency (Baumeister et al., 1994). During the COVID-19 pandemic, nurses need to wear protective tools when performing nursing operations, which not only brings difficulty and danger to completing nursing operations but also places nurses in a state of stress for a long time, resulting in nurses’ ego depletion. In addition, due to closed management, nurses also have to take care of patients’ lives, which greatly increases work intensity and consumes nurses’ psychological resources (Riedel et al., 2021). Data from several studies have suggested that the more serious ego depletion is, the stronger the sense of work alienation (Zhao and Liu, 2019). With an increase in ego depletion, employees will reduce their work commitment and exhibit uncivilized, deviant, and anti-productive behaviors in the workplace (Ma et al., 2020). Thus, limiting ego depletion as much as possible is essential to prevent work alienation. Indeed, empathy can help nurses improve their work autonomy, recognize the value of work, put more energy into work, effectively alleviate the consumption of self-control resources, and reduce ego depletion (Martos Martínez et al., 2021). Hence, we proposed Hypothesis 2: Ego depletion mediates the association between empathy and work alienation.

**Figure 1:**
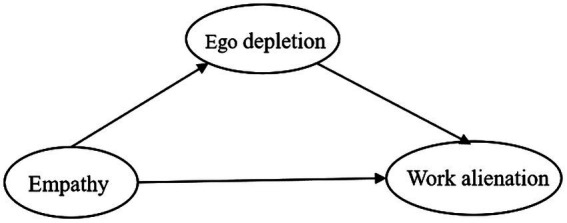
Hypothesis model of empathy as a predictor of work alienation mediated by ego depletion.

Based on the literature, empathy, ego depletion, and work alienation are correlated with each other, but the mechanism of empathy on work alienation among Chinese nurses lacks attention. Therefore, we proposed a hypothetical model for this study, which is shown in Figure 1. In the current study, we aimed to analyze the associations among empathy, ego depletion, and work alienation of Chinese nurses during the COVID-19 pandemic and to further explore the mechanism by which empathy influences work alienation to provide a theoretical basis and guidance for reducing nurses’ work alienation and stabilizing nursing teams.

## 2. Materials and methods

### 2.1. Design and participants

This study used a cross-sectional survey design. A total of 353 registered nurses from three grade 3A hospitals in Xi’an, Shaanxi Province, China, were selected to participate in the study by the convenience sampling method from June 2021 to October 2021. The primary inclusion criteria were nurses engaged in clinical nursing work who had been working for at least 1 year and nurses who gave informed consent and voluntarily participated in this study. The exclusion criteria were advanced students, as well as nurses who were on sick leave or maternity leave.

The sample size was calculated according to the principle of Kendall estimation of sample size (Ni et al., 2010). This demonstrated that the sample size was 5~10 times that of the independent variables. There were 20 independent variables in this study. Considering a 20% sample loss rate, the minimum sample size was [20 × 5 × (1 + 20%)] = 120. On the other hand, Mueller (1997) proposed that the sample size of the structural equation model should be more than 200. Hence, a random sample of 353 nurses was included in this study.

### 2.2. Measures

#### 2.2.1. General demographic data

The general demographic data collected by the researchers, included gender, age, years of working experience, department, education background, personnel status, marital status, child/children, technical title, job role, and monthly income.

#### 2.2.2. Empathy

The Jefferson Scale of Empathy-Health Professionals (JSE-HP) assessed nurses’ empathy ability. It was developed by Hojat et al. (2002) of the Medical Education and Health Care Research Center from Jefferson University in the United States; it was translated into Chinese and tested for reliability and validity by the Chinese scholar An et al. (2008). The scale consists of 20 items in three dimensions: perspective-taking (10 items), compassionate care (8 items), and standing in the patients’ shoes (2 items). Each item is scored on a Likert scale ranging from completely disagree (1) to completely agree (7), with a total score of 20 to 140. The higher the score was, the higher the nurses’ empathy. A total empathy score ≤ 60 indicates a low level, 61 to 99 indicates a moderate level and ≥100 indicates a high level. The Cronbach’s α of the total scale in this study was 0.862.

#### 2.2.3. Ego depletion

The Self-Regulating Fatigue Scale (SRF-S) measures the state of nurses’ trait ego depletion. It was compiled by Nes et al. (2013) from Australia and translated into Chinese and revised by Wang et al. (2015). The scale consists of 16 items in three dimensions: cognition (6 items), emotion (5 items), and behavior (5 items). Each item uses the Likert 5-point scoring method, which ranges from strongly disagree (1) to strongly agree (5). The higher the score was, the more serious the individual’s ego depletion. The Cronbach’s α of the total scale in this study was 0.842.

#### 2.2.4. Work alienation

The work alienation questionnaire was developed by Chinese scholar Ren (2012) and includes 12 items in three dimensions: helplessness, friendlessness, and meaninglessness. Each item is answered on a 5-point Likert scale from strongly disagree (1) to strongly agree (5), with a higher score indicating higher work alienation. The Cronbach’s α of the total scale in this study was 0.883.

### 2.3. Ethical considerations and data collection

This study was approved by the Ethics Committee of the Second Affiliated Hospital of Air Force Medical University (No. 202206-02) and conducted under ethical guidelines described in the Helsinki Declaration (World Medical Association 2013). The data were collected anonymously using an online questionnaire *via* Wenjuanxing.^1^ The questionnaire used unified instructions to explain the purpose, significance, and expected completion time of the survey to participants and asked for their verbal consent. Before sending out the questionnaire, we designated a responsible person in each hospital and trained him to fully understand the purpose and inclusion criteria of the survey, and he then mobilized, guided, and supervised the nurses in his hospital to complete the questionnaire. All questions were compulsory, and the questionnaire could be submitted only after all questions had been answered. During the investigation, participants could withdraw at any time. Ultimately, a total of 353 questionnaires were collected online.

### 2.4. Data analysis

The data were input into Epidata 3.1 and analyzed by SPSS for Windows Version 26 and Mplus 8.3. SPSS 26.0 was used for common method bias, descriptive statistical analysis and correlation analysis. The mean, standard deviation (SD), frequency, and percentage were used to present the descriptive data. The Kolmogorov–Smirnov (K-S) test and skewness and kurtosis tests were used to examine the normality of all variables in our study. All the data were normally distributed. Furthermore, we explored the correlation among empathy, ego depletion, and work alienation by using Pearson’s correlation coefficient (r). A structural equation model was constructed using Mplus 8.3 software to analyze the mechanism of empathy and ego depletion on work alienation. Among them, empathy was an independent variable, ego depletion was a mediating variable, and work alienation was a dependent variable. The maximum likelihood method was used to evaluate the measurement model, and the bootstrap method was used to evaluate the structural model (bootstrap = 1,000). If the 95% confidence interval (CI) of the indirect effect did not contain zero, then the mediating effect was considered significant. We set the statistical significance level at 0.05 for all analyses (*p* < 0.05).

## 3. Results

### 3.1. Common method bias

To test common method bias, we performed the Harman univariate test. All variables were put into an exploratory factor analysis to determine the fewest factors necessary to explain the variation in variables. If only one factor was separated out or the explanatory power of a factor was particularly large, we assumed a serious common method bias. In our study, 9 factors were separated out, and the variable interpretation rate of the first factor was 23.644%, less than 40%, suggesting that there was no common method bias.

### 3.2. Nurse characteristics

A total of 353 nurses were involved in our study, with an average age of 29.93 ± 6.25 (range = 19–61). The majority were female (*n* = 331, 93.77%) and had worked only for 1 year (*n* = 250, 70.82%). The detailed characteristics of the nurses are presented in Table 1.

**Table 1:** Nurse characteristics (*n* = 353).

Variables	*n* (%)	Variables	*n* (%)
**Gender**		**Personnel status**	
Male	22(6.23)	Formal nursing staff	43(12.18)
Female	331(93.77)	Hires nurse	310(87.82)
**Age**		**Marital status**	
18~	194(54.96)	Married	196(55.52)
30~	134(37.96)	Single	153(43.34)
40~	21(5.95)	Divorced	2(0.57)
50~	4(1.13)	others	2(0.57)
**Years of work experience**		**Children’s status**	
1~	250(70.82)	No children	197(55.81)
11~	89(25.21)	One child	113(32.01)
21~	10(2.83)	Two children	42(11.90)
31~	4(1.13)	Three children or more	1(0.28)
**Department**		**Technical title**	
Gastroenterology	37(10.48)	Nurse	94(26.63)
Surgery	18(5.10)	Senior nurse	162(45.89)
Obstetrics-gynecology	32(9.07)	Supervisor nurse	93(26.35)
Pediatrics	56(15.86)	Deputy director nurse and above	4(1.13)
Operating room	15(4.25)	**Job role**	
Intensive Care Unit	39(11.05)	None	327(92.63)
Others	156(44.19)	Nurse manager	26(7.37)
**Education background**		**Monthly income (CNY)**	
Polytechnic school	3(0.85)	2,000~	97(27.48)
Junior college	81(22.95)	4,000~	140(39.66)
Bachelor’s	261(73.94)	6,000~	61(17.28)
Master’s	7(1.98)	8,000~	43(12.18)
Doctorate	1(0.28)	10,000~	12(3.40)

### 3.3. Descriptive statistics of empathy, ego depletion, and work alienation

The mean score for empathy was 109.17 (SD: 14.52); 80 nurses (22.66%) scored 61–99, which was a moderate level, while 273 nurses (77.34%) scored ≥100, which was a high level. The mean scores for ego depletion and work alienation were 41.89 (SD:8.66) and 30.90 (SD:8.27), respectively. Table 2 shows the total score and the scores for each dimension: empathy, ego depletion, and work alienation.

**Table 2:** Descriptive summary of nurses’ empathy, ego depletion and work alienation (*n* = 353).

Variable	Number of items (*n*)	Range	Mean ± SD ($\bar{x}$*± s*)	Mean ± SD ($\bar{x}$*± s*) (each item)
Total score of empathy	20	20–140	109.17 ± 14.25	5.46 ± 0.71
Perspective-taking	10	10–70	54.07 ± 8.79	5.41 ± 0.88
Compassionate care	8	8–56	44.03 ± 7.50	5.50 ± 0.94
Standing in the patients’ shoes	2	2–14	11.07 ± 2.17	5.53 ± 1.08
Total score of ego depletion	16	16–80	41.89 ± 8.66	2.62 ± 0.54
Cognition	6	6–30	17.26 ± 2.72	2.88 ± 0.45
Emotion	5	5–25	12.91 ± 3.49	2.58 ± 0.70
Behavior	5	5–25	11.72 ± 3.69	2.34 ± 0.74
Total score of work alienation	12	12–60	30.90 ± 8.27	2.58 ± 0.69
Helplessness	4	4–20	12.76 ± 3.23	3.19 ± 0.81
Friendlessness	4	4–20	8.88 ± 3.19	2.22 ± 0.80
Meaninglessness	4	4–20	9.26 ± 3.56	2.32 ± 0.89

### 3.4. Correlation between nurses’ empathy, ego depletion, and work alienation

The Pearson correlation analysis showed that work alienation was negatively correlated with empathy (*r* = −0.305, *p* < 0.01) and positively correlated with ego depletion (*r* = 0.652, *p* < 0.01). Empathy was negatively correlated with ego depletion (*r* = 0.325, *p* < 0.01).

### 3.5. Mediating effect of ego depletion on the association between empathy and work alienation

**Figure 2:**
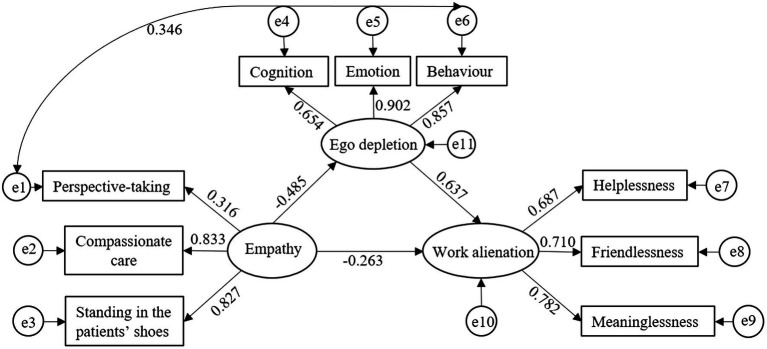
A structural equation model of the mediating effect of ego depletion on the relationship between nurses’ empathy and work alienation. All factor loadings were significant at the *p* < 0.05 level.

Empathy was the independent variable, while perspective-taking, compassionate care, and standing in the patients’ shoes were the latent variable indexes of empathy. Ego depletion was the mediating variable, while cognition, emotion, and behavior were the latent variable indexes of ego depletion. Work alienation was the dependent variable, while helplessness, friendlessness, and meaninglessness were the latent variable indexes of work alienation. We used Mplus 8.3 software to construct the structural equation model, as shown in Figure 2.

The maximum likelihood method was used to fit the model, which was modified according to the modified index of the model. After adding one path, the fitting results of the model are shown in Table 3. All fitting indexes were in the acceptable range, indicating that the fitting degree of the model was good and that the model could be accepted. The results showed that empathy had a direct negative predictive effect on work alienation (*β* = −0.263, *p* < 0.01). Moreover, empathy had a negative predictive effect on ego depletion (*β* = −0.485, *p* < 0.01), and ego depletion had a positive predictive effect on work alienation (*β* = 0.637, *p* < 0.01). The direct effect, indirect effect, and total effect of empathy on work alienation are shown in Table 4. The mediating effect of ego depletion was −0.309, accounting for 54.02% of the total effect (−0.572).

**Table 3:** Fitting indexes of the mediating effect model.

Fitting index	*χ^2^*	*df*	*χ^2^/df*	*RMSEA*	*CFI*	*TLI*	*SRMR*
Model index	68.388	23	2.97	0.075	0.966	0.947	0.042

df, degrees of freedom; RMSEA, Root Mean Square Error of Approximation; CFI, Comparative Fit Index; TLI, Tucker-Lewis Index; SRMR, Standardized Root Mean Square Residual.

**Table 4:** Direct effect, indirect and total effect of variables (standardized).

Independent variable	Dependent variable	Direct effect	Indirect effect	Total effect
Empathy	Work alienation	−0.263	−0.309	−0.572
Empathy	Ego depletion	−0.485	0	−0.485
Ego depletion	Work alienation	0.637	0	0.637

We then used the bootstrap method to test the significance of the mediating effect. The sample size was set to 1,000 (number of iterations), and the confidence interval was set to 95%. If the 95% confidence interval did not contain zero, the mediating effect was significant. The results showed that the 95% confidence intervals of the standardized direct effect and mediating effect were [−0.396, −0.132] and [−0.406, −0.226], respectively, which did not contain zero, indicating that the partial mediating effect was significant. In other words, the mediating effect model of ego depletion on empathy and work alienation was established.

## 4. Discussion

In general, the results of this study evaluate the current situation of Chinese nurses’ empathy, ego depletion, and work alienation and show that empathy has a significant direct and indirect effect on work alienation. These findings confirm the hypotheses.

This study showed that nurses’ empathy was at the moderate level, which was lower than that of the Spanish nurses studied by Yuguero et al. (2018), which indicates that there is much room for improvement in the empathy ability of nurses in China. Among the indicators of empathy, perspective-taking had the highest mean score, followed by compassionate care, and standing in the patients’ shoes. China has been strengthening the cultivation of the empathy ability of nursing students and clinical nurses in recent years, according to the literature; however, most of the educational content is about how to stand in the patients’ shoes, with little mentioned about compassionate care and perspective-taking (Wu, 2021). In the clinical work of nurses, the concept of ‘patient-centered practices’ often emphasizes the importance of standing in the patients’ shoes, so most nurses seem to think that it is the most important way to express empathy (Lee and Ihm, 2021). When nurses thought about problems from a patient’s point of view, they could take care of patients’ emotions, comfort and encourage them, and provide compassionate care. However, perspective-taking requires nurses to understand and accept patients’ viewpoints, which is relatively difficult.

Nurses’ ego depletion was also moderate. The three dimensions in order of mean score were cognition, emotion, and behavior, which was consistent with the research results of Zhang et al. (2021). This showed that nurses had serious ego depletion, which should be considered by nursing managers. Nursers are the main force in the rescue team on the front line of fighting against the COVID-19 pandemic. In isolation wards, nurses not only bear a heavy workload but also face the constant risk of infection. According to the theory of limited resources of self-control put forward by Baumeister et al. (1998), nurses need to initiate cognitive, emotional, and behavioral control independently in the face of heavy work tasks, pressure, tense doctor-patient relationships, and so on. Prolonged self-control consumes many psychological resources and energy, destroys the energy balance, and thus causes ego depletion (Heckman et al., 2017).

The score of nurses’ work alienation was higher than the results of Jiang et al. (2020) study on nurses in a dialysis department, and indicated that nurses’ work alienation was increasing. Moreover, most of the nurses in our study only worked for 1 year, so the serious sense of work alienation in such a short period of time is worth our attention. This may be due to the continuous nature of the virus during the COVID-19 pandemic and the repeated waves of the disease, which substantially increased nurses’ workload. In addition to finishing their routine nursing work, the nurses also needed to complete additional tasks, such as nucleic acid testing and epidemic prevention and control. At the same time, the risk of contracting COVID-19 at work also increased; thus, nurses tended to have negative emotions about work (Chatzittofis et al., 2021). The long-term accumulation of negative emotions led to a continuous decrease in nurses’ expectations and satisfaction with work (Modaresnezhad et al., 2021), and finally, there was a sense of work alienation.

The current study also demonstrated that the higher the level of nurses’ empathy was, the less ego depletion and work alienation. The higher the level of ego depletion was, the stronger the work alienation. Our results supported previous studies that showed that empathy was negatively correlated with ego depletion and work alienation, while ego depletion was positively correlated with work alienation (Finley et al., 2017; Delgado Bolton et al., 2022). Nurses with high empathy could correctly perceive their own emotions and those of their patients, reasonably and effectively regulate their cognition, emotion, and behavior, reduce their energy consumption, get along well with patients, and experience a better sense of value and achievement in their work. Over time, the sense of work alienation also diminished. However, nurses with high ego depletion had a decline in self-control. Some studies have shown that when the input and expenditure of individual psychological energy are unbalanced, the results are a loss of self-control and the expression of negative emotions (Xue, 2020), interpersonal conflicts (Gotaas et al., 2021), workplace violence (Roczniewska and Bakker, 2021), and negligent work behaviors (Majani et al., 2016). Psychological resources are constantly consumed, which eventually leads to an increasing sense of work alienation.

Mediation analysis showed that ego depletion played a partial mediating role in the relationship between nurses’ empathy and work alienation. That is, empathy not only had a direct negative predictive effect on work alienation but also used ego depletion as a mediating variable to negatively and indirectly predict work alienation. Empathy is a core element of nursing work. Nurses have professional medical knowledge, and some nurses are even infected with COVID-19, so they are more able to think from the patients’ points of view. A high level of empathy helps to establish a good nurse–patient relationship, which not only helps patients but also increases nurses’ professional identity. Self-affirmation at work can supplement nurses’ energy and reduce the consumption of self-control energy and ego depletion (Storr and Sparks, 2016). In addition, Nowrouzi et al. (2015) found that empathy helped to create a relaxed and comfortable working atmosphere and provided nurses with positive emotional resources and a healthy working environment, which may be crucial in reducing work alienation. Empathy could also improve an individual’s positive emotions, and a high level of empathy was more likely to increase happiness (Sugimori et al., 2020). In clinical practice, nurses with a high level of empathy experienced more positive emotions such as satisfaction and pleasure after helping others, while the construction of positive emotions could supplement their psychological resources and significantly reduce ego depletion, thus reducing nurses’ work alienation. We can improve nurses’ empathy through theoretical study and clinical practice. At the same time, nursing managers should optimize the nursing workflow, continuously improve the scheduling system, and maximize the rest time of front-line nurses, which is the most direct and effective way to supplement the psychological energy of nurses.

There are limitations to this study. First, there may be large individual differences among the participants because of the convenience sampling method. Second, only 353 Chinese nurses were included in this study, so the sample size was not very large. We should expand the sample size and carry out research in different regions and hospitals at all levels to obtain more reliable data. Third, the questionnaires we used in the study were self-reported, so the responses were subjective and might produce bias. We can try to measure neurological or endocrine indicators to verify the relationship between physiological indicators and psychological problems in the future. Finally, in the analysis of influencing factors, the sociodemographic data of the samples were not discussed and need to be explored in future research. This will allow us to determine what characteristics of nurses are likely to lead to the strongest sense of work alienation, and then we can provide targeted interventions based on this information.

## 5. Conclusion

This study has identified that empathy has a negative predictive effect on ego depletion and work alienation and that ego depletion has a positive effect on work alienation. We enrich the mechanistic research on work alienation and confirm the mediating effect of ego depletion on the relationship between empathy and work alienation among Chinese nurses. The current results highlight the importance of improving empathy and reducing ego depletion to prevent the occurrence of work alienation.

## 6. Relevance for clinical practice

We find that some nurses have a serious sense of work alienation during the COVID-19 pandemic, which may induce a series of serious consequences, such as an increase in the turnover rate, the occurrence of nursing safety incidents, and a decline in nursing quality. Therefore, nursing managers should pay attention to nurses’ work alienation and discover and intervene as soon as possible. Through the elucidation of the mediating effect model, nursing managers can intervene in the following two ways. First, nursing managers should directly train nurses’ empathy abilities through role exchange training courses (Zhu et al., 2021) and can also raise nurses’ consciousness through interventions such as mindfulness therapy, narrative nursing, and Balint groups to reduce work alienation. Nursing managers can also start with the aim of reducing nurses’ ego depletion, strengthening nurses’ social support systems, constructing positive psychological resources, improving scheduling to ensure adequate rest after the night shift, inspiring nurses’ self-affirmation, and enhancing nurses’ patient-centered work motivation. They should also listen to nurses, relieve their work pressure, help them recover from ego depletion, and reduce work alienation.

## Data availability statement

The raw data supporting the conclusions of this article will be made available by the authors, without undue reservation.

## Ethics statement

The studies involving human participants were reviewed and approved by Ethics Committee of the Second Affiliated Hospital of Air Force Medical University (No. 202206-02). The patients/participants provided their written informed consent to participate in this study.

## Author contributions

YC and YZ designed the research. YC contributed to writing all parts of the manuscript. HY, MZ, and NL were responsible for collecting the data. TY, QL, and LiZ contributed to the data analysis. YZ and LaZ reviewed the manuscript and made constructive suggestions. All authors contributed to the article and approved the submitted version.

## Funding

This research was supported by the Military Medical ‘Everest Project’ boost fund of Air Force Medical University (2020ZFB012).

## Conflict of interest

The authors declare that the research was conducted in the absence of any commercial or financial relationships that could be construed as a potential conflict of interest.

## Publisher’s note

All claims expressed in this article are solely those of the authors and do not necessarily represent those of their affiliated organizations, or those of the publisher, the editors and the reviewers. Any product that may be evaluated in this article, or claim that may be made by its manufacturer, is not guaranteed or endorsed by the publisher.

## Footnotes

^1^ http://www.wjx.cn/
